# Advantages of a Mechanistic Codon Substitution Model for Evolutionary Analysis of Protein-Coding Sequences

**DOI:** 10.1371/journal.pone.0028892

**Published:** 2011-12-29

**Authors:** Sanzo Miyazawa

**Affiliations:** Graduate School of Engineering, Gunma University, Kiryu, Gunma, Japan; Institute of Infectious Disease and Molecular Medicine, South Africa

## Abstract

**Background:**

A mechanistic codon substitution model, in which each codon substitution rate is proportional to the product of a codon mutation rate and the average fixation probability depending on the type of amino acid replacement, has advantages over nucleotide, amino acid, and empirical codon substitution models in evolutionary analysis of protein-coding sequences. It can approximate a wide range of codon substitution processes. If no selection pressure on amino acids is taken into account, it will become equivalent to a nucleotide substitution model. If mutation rates are assumed not to depend on the codon type, then it will become essentially equivalent to an amino acid substitution model. Mutation at the nucleotide level and selection at the amino acid level can be separately evaluated.

**Results:**

The present scheme for single nucleotide mutations is equivalent to the general time-reversible model, but multiple nucleotide changes in infinitesimal time are allowed. Selective constraints on the respective types of amino acid replacements are tailored to each gene in a linear function of a given estimate of selective constraints. Their good estimates are those calculated by maximizing the respective likelihoods of empirical amino acid or codon substitution frequency matrices. Akaike and Bayesian information criteria indicate that the present model performs far better than the other substitution models for all five phylogenetic trees of highly-divergent to highly-homologous sequences of chloroplast, mitochondrial, and nuclear genes. It is also shown that multiple nucleotide changes in infinitesimal time are significant in long branches, although they may be caused by compensatory substitutions or other mechanisms. The variation of selective constraint over sites fits the datasets significantly better than variable mutation rates, except for 10 slow-evolving nuclear genes of 10 mammals. An critical finding for phylogenetic analysis is that assuming variable mutation rates over sites lead to the overestimation of branch lengths.

## Introduction

Growing DNA and protein sequence data is now a valuable source of knowledge in many fields of science, especially in evolutionary biology. Evolutionary history of DNA sequences is a key to understand the diversity of homologous sequences. Any method for inferring molecular phylogeny is implicitly or explicitly based on the evolutionary model of nucleotide or amino acid substitutions, and the reliability of phylogenetic analyses strongly depends on models designed to approximate the substitution processes of nucleotide and amino acid. For the evolutionary analysis of protein-coding sequences, three types of models can be used: nucleotide, amino acid, and codon substitution models. Which type of model fits any sequence data better than the others? Mutational events occur at the nucleotide level, but selective pressure primarily operates at the amino acid level. Thus, a codon substitution model has a potential to outperform both nucleotide substitution models [1]–[3] and amino acid substitution models [4]–[12], because it can take into account both mutational tendencies at the nucleotide level and selective pressure on amino acid replacements as well as a genetic code. Shapiro et al. [13] proposed a codon position model, in which codon position is incorporated into a nucleotide substitution model. This model is computationally efficient but insufficient to take account of the dependencies of selective pressure on amino acid replacements.

Codon substitution models are classified into either an empirical codon substitution model or a mechanistic codon substitution model. In empirical codon substitution models [14], [15], substitution rates between codons were empirically estimated from a large set of protein-coding sequences, and mutational tendencies at the nucleotide level and selection pressure at the amino acid level cannot be separated at all. Therefore, there is no parameter except codon frequencies to tailor for each protein family. Delport et al. [16] showed that empirical substitution matrices represent the average tendencies of substitutions over various protein families by sacrificing gene-level resolution.

In mechanistic codon substitution models, a mutational mechanism at the nucleotide level and selection at the amino acid level are distinguished in various levels of separation. If no selection pressure on amino acids is taken into account, the codon substitution model will become essentially equivalent to a nucleotide substitution model. If mutation rates are assumed not to depend on the codon type, then the model will become essentially equivalent to an amino acid substitution model. Such a codon model with the infinitely large synonymous substitution rate, was proved [17] to be exactly equivalent to an amino acid substitution model. It was shown on protein-coding sequences that codon substitution models are statistically superior to the nucleotide and amino acid substitution models [18], [19].

There are two type of models for the mutational scheme of codon, depending on whether multiple nucleotide changes in infinitesimal time are allowed [17], [19]–[21] or not. Even though all the empirical amino acid substitution models [4]–[6], [8], [10], [11] and the empirical codon substitution model [15] allow amino acid or codon substitutions requiring multiple nucleotide changes in infinitesimal time, only single nucleotide changes were assumed to occur in infinitesimal time [7], [18], [22]–[25]. Multiple nucleotide changes in infinitesimal time are biologically plausible, because they can be caused by successive compensatory substitutions [26], recombination, gene conversion and other mechanisms [27], especially in long branches. It has been pointed out that assuming multiple nucleotide changes in codon substitution models can significantly improve the maximum likelihood (ML) value [19], [20].

In the present models, mutational tendencies at the nucleotide level are tailored to each gene by the general time-reversible (GTR) model, but multiple nucleotide changes in infinitesimal time are allowed. In the Singlet-Doublet-Triplet (SDT) mutation model [20], single-nucleotide, doublet and triplet mutations spanning codon boundaries are taken into account, but double nucleotide mutations at the first and the third positions in a codon were not taken into account. In the present model, it is assumed [19] that nucleotide mutations occur independently at each codon position and so any double nucleotide mutation occurs as frequently as doublet mutations.

There are a variety of models for selection pressure on amino acid replacements in mechanistic codon substitution models; (1) models [17], [18], [21] based on empirical amino acid substitution matrices, in which codon exchangeabilities for nonsynonymous substitutions were evaluated on the basis of empirical amino acid exchangeabilities, and selective constraints on amino acids are not well separated from codon mutation rates, (2) equal-constraint models [20], [24], [28], [29], in which the difference between nonsynonymous and synonymous substitution rates was taken into account but the amino acid dependences of selective constraints on amino acids were not taken into account, i.e., single selective constraints for all types of amino acid substitutions, (3) physico-chemical-constraint models [7], [22], [23], in which selective constraints for each protein family were approximated in a linear function of the selective constraints evaluated from physico-chemical properties of amino acids, (4) fully-parameterized-constraint models [7], [16], [25], in which selective constraints were grouped, and the number of groups and the strength of selective constraint of each group were optimized for a given protein phylogeny, and (5) site-specific selection models [30], in which site-specific selection was modeled in terms of site-specific residue frequencies in a codon substitution model.

In the models [17], [21] of the first category, codon exchangeabilities for nonsynonymous codon substitutions requiring multiple nucleotide changes are set to non-zero according to the empirical amino acid exchangeabilities; the exchangeability is defined to be an instantaneous rate divided by the equilibrium composition of destination codon or amino acid. The method in the fourth category has the highest resolution of selective constraints employing as many substitution groups as necessary. However, it seems to be a very computer-intensive calculation [25].

In the present model, selective constraints on the respective types of amino acid replacements are tailored to each gene in a linear function of a given estimate of selective constraints in the same way with the physico-chemical-constraint models. The simplest model for the selective constraints is to assume equal constraint on amino acid replacements and equivalent to the second category of model; it is named here the Equal-Constraint model. Of course, physico-chemical estimates of the selective constraints can also be used [19]. Better estimates are those that were estimated [19] by maximizing the respective likelihoods of observed amino acid or codon substitution frequency matrices.

A property of codon substitution models in which synonymous substitutions can be identified is an advantage over nucleotide and amino acid substitution models. Significance of rate variation over sites in proteins has been demonstrated mostly in nucleotide substitution models and empirical amino acid substitution models [31]–[33]. Variable rates of nucleotide and amino acid substitutions over sites can be caused not only by the variation of mutation rate but also by the variation of selective constraint over sites. However, in the nucleotide and the amino acid substitution models, synonymous substitutions cannot be recognized, and therefore the variations of mutation rate and of selective constraint over sites cannot be distinguished from each other. On the other hand, the variations of selective constraint and mutation rate can be distinguished from each other in codon substitution models, assuming no selective pressure on synonymous mutations at the amino acid level. It is reasonable from a viewpoint of protein structure and function that amino acid replaceabilities strongly depend on sites in a protein [34]. Molecular mechanisms are not known to cause significantly variable mutation rates over sites within the exons of a gene. Here, we examine which model fits data statistically better. In the present model, either the variation of mutation rate or the variation of selective constraint is taken into account, although both is not taken into account at the same time because of heavy computational load. Yang et al. [29] also studied heterogeneous selection pressure at amino acid sites by codon substitution models.

Besides the variation of substitution rate over sites, the variation of substitution rate over time at each site is also possible. The site-specific variation of substitution rate over time was first discussed as a covarion model by Fitch and Markowitz [35]. Recently, a few cases indicating its significance have been reported [36], [37]. Here we take into account the variation of mutation rate over time at each site in a simple approximation.

The estimation of branch lengths is critical on the estimation of phylogeny and divergence times. We examine how differently branch lengths are estimated between models. The present mechanistic codon substitution model can simulate a wide range of codon substitution processes by changing parameters, and can provide biologically meaningful information at both nucleotide and amino acid levels such as transition/transversion rate bias, the ratio of multiple nucleotide changes, the strength of selective constraints on amino acids, the variation of mutation rate or selective constraints over sites, and also the variation of mutation rate over time in branches. Here, the present codon substitution models with the various sets of parameters are extensively studied, and the advantages of the present model over other models are demonstrated.

## Methods

### A time-reversible Markov model for substitutions

When substitutions independently occur at each site with a constant substitution rate 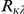 per unit time from codon or amino acid 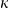 to 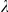, the substitution probability matrix 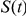 at time 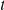 is calculated as
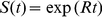
(1)Assuming that the detailed balance condition between states is satisfied, i.e., 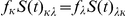 and 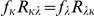, the substitution rate 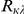 is represented as
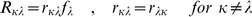
(2)where 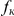 is the equilibrium composition; 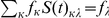. The symmetric matrix 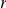 is named an exchangeability matrix. In the case of the codon substitution matrix, the equilibrium frequencies of stop codons are set to be equal to 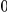, and therefore the probability flow from any to a stop codon and its inverse flow are always equal to 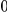. The unit of time is chosen in such a way that the total rate of 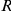 is equal to 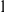;
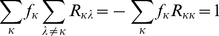
(3)Therefore, only the relative values among 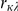 are meaningful.

In a given phylogeny of molecular sequences, a substitution process of codon or amino acid is assumed to be in equilibrium. In other words, the substitution process is assumed to be time-reversible. Also, exchangeabilities 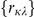 are approximated not to depend on the equilibrium frequencies 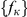; this model is specified here with a suffix “F” according to a common naming convention.

### Empirical amino acid substitution models converted into codon substitution models

Amino acid exchangeabilities 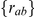 for amino acid substitutions have been estimated from large sets of protein sequences. From nuclear proteins, the JTT [5], the WAG [10], and the LG [11] rate matrices were estimated. The mtREV [6] substitution probability matrix was estimated from vertebrate mitochondrial proteins, and the cpREV10 [8] and the cpREV64 [38] matrices were estimated from chloroplast proteins of 10 species and of 64 species, respectively.

These amino acid substitution models can be converted into codon substitution models by defining codon exchangeabilities on the basis of amino acid exchangeabilities between encoded amino acids [17]–[19], [21]. Here we consider the following simplest conversion from the amino acid models into codon models to examine the performance of the empirical amino acid substitution models in phylogenetic inference from coding sequences.

The codon exchangeability 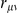 between nonsynonymous codons 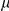 and 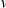 is defined to be proportional to the empirical amino acid exchangeability 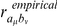 between encoded amino acids 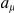 and 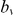 with a parameter 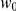 to adjust the ratio of synonymous to non-synonymous substitution exchangeability. Codon exchangeabilities between synonymous codons are taken to be all equal to one another in such a way that in the case of 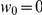 they are equal to the maximum exchangeability of nonsynonymous substitutions.
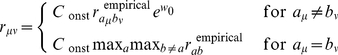
(4)where 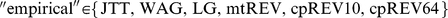. The arbitrary scaling constant 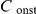 is determined by Eq. 3. In the limit of 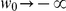, this model is exactly equivalent to the corresponding amino acid substitution model [17]. This model was named as the SK-P1 model by Seo and Kishino [17], and is called here by the name of the empirical amino acid substitution matrix with a suffix meaning the number of ML parameters such as JTT-*n*, WAG-*n*, LG-*n*, mtREV-*n*, cpREV10-*n*, and cpREV64-*n*.

### Empirical codon substitution models

Kosiol et al. [15] estimated codon exchangeabilities 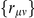 from nuclear-encoded sequences; this substitution rate matrix is called here the KHG matrix. This empirical codon substitution model has been extended here with a parameter 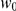 to adjust the ratio of synonymous to non-synonymous substitution exchangeability.
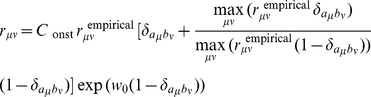
(5)for 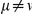, where 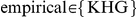, and 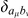 is the Kronecker's 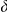. The arbitrary scaling constant 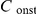 is determined by Eq. 3. The exchangeabilities of nonsynonymous codon substitutions are scaled in such a way that in the case of 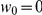 the maximum exchangeability of nonsynonymous substitutions is equal to that of synonymous substitutions. This model is called KHG-*n*, where the suffix *n* means the number of ML parameters.

### A mechanistic codon substitution model with multiple nucleotide changes

In the present mechanistic codon substitution model [19], the substitution rate 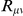 is represented as the product of a mutation rate 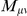 and the average rate of fixation 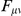, which is defined to be the average fixation probability multiplied by the chromosomal population size, for mutations from codon 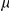 to 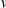 under selection pressure; 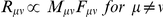. The 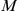 is also assumed to satisfy the detailed balance condition; 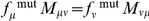, where 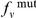 is the equilibrium codon composition of the rate matrix 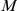. Under this assumption, the average fixation rate 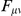 must be represented as the product of the two terms, 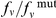 and 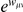, where 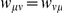; 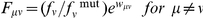. Then, the exchangeability 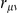 can be represented as
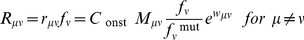
(6)The arbitrary scaling constant 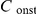 is is determined by Eq. 3.

The frequency-dependent term 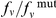 represents the effects of selection pressures at the DNA level as well as at the amino acid level, which change the codon frequency from the mutational equilibrium frequency 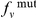 to the frequency 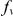 specific to a gene. The fixation rate 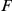 was explicitly given as a function of the fitnesses of mutants 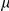 and 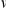 [28], [30]. The fixation rate is obviously equal to 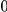 for lethal mutations and equal to 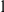 for neutral mutations. Here, we approximate the average quantity 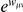 over mutants to be independent of codon frequencies. This quantity 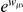 is essentially the same as the one called the rate of acceptance by Miyata et al. [39]. We assume that selection pressure against codon replacements appears primarily on an amino acid sequence encoded by a nucleotide sequence; in other words, 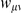 for codon pair 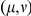 is equal to the selective constraint 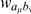 for the encoded amino acid pair 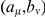.
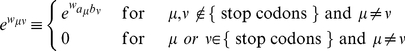
(7)At the amino acid level, there should be no selection pressure against synonymous mutations. Thus, the 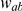 satisfies
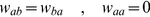
(8)


Selective constraints 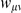 are evaluated for a specific protein family in a linear function of a given estimate of 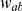;
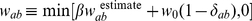
(9)where 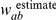 with 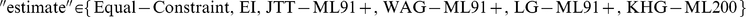 means the estimate of 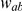, which is equal constraint on amino acids (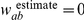 or 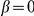), a physico-chemical estimate based on the Energy-Increment-based (EI) method [19], or a ML estimate [19] from the empirical substitution frequency matrix of JTT, WAG, LG, or KHG. The value of 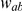 is non-positive, assuming that on average there is negative selection on amino acid replacements; of course, 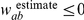 [19]. The parameter 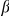, which is non-negative, adjusts the strength of selective constraints for a given protein family. The parameter 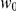 directly controls the ratio of nonsynonymous to synonymous substitution exchangeability. Positive selection is taken into account when selective constraints are variable over sites.

The Equal-Constraint model with 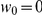 is called the No-Constraint model and is equivalent to a nucleotide substitution model. In the model EI, 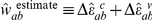, where 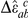 and 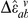 represent the mean increment of contact energies between residues and the mean volume change due to an amino acid replacement, respectively; see Supporting Information, Text S1, in [19]. The selective constraint matrices 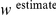 with 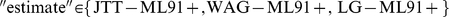 were those estimated by maximizing the respective likelihoods of the 1-PAM amino acid substitution frequency matrices of JTT, WAG, and LG in the ML-91+ model [19]. Similarly, the matrix 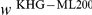 were estimated from the 1-PAM KHG codon substitution frequency matrix in the ML-200 model [19]. These estimates of selective constraints are available as Supporting Information, Data S1, in [19]. These models are called here by the name of a selective constraint matrix with a suffix meaning the number of ML parameters such as Equal-Constraint-*n*, EI-*n*, JTT/WAG/LG-ML91+-*n*, and KHG-ML200-*n*.

The mutation rate matrix 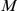 is defined in terms of nucleotide mutation rates as follows.
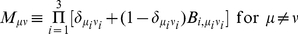
(10)where 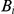 is a mutation rate matrix between the four types of nucleotides at the 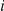
th codon position, 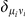 is the Kronecker's 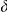, and the index 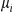 means the 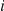
th nucleotide in the codon 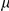; 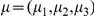 where 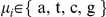. Assuming that the rate matrix 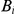 satisfies the detailed balance condition, it is represented as
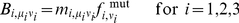
(11)

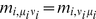
(12)

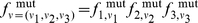
(13)where 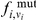 is the equilibrium composition of nucleotide 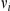 at the 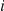
th codon position, and 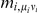 is the exchangeability between nucleotides 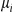 and 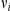 at the 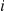
th codon position. Because the 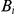 is assumed to satisfy the detailed balance condition, the 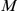 also satisfies the detailed balance condition.

If multiple nucleotide changes were completely ignored, then Eq. 10 would be simplified as 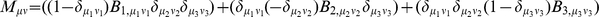, whose formulation for a codon mutation rate matrix with Eq. 11 is the same as the one proposed by Muse and Gault [24]. Here, it should be noted that 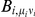 in Eq. 11 is defined to be proportional to the equilibrium nucleotide composition 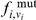. Alternatively, one may define 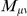 as 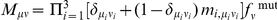 in the same way as Miyazawa and Jernigan [22] and others [7], [23] defined it to be proportional explicitly to the composition of the base triplet, 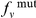. This alternative definition with Eqs. 6 is equivalent to Eqs. 10 and 11 with 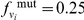 and 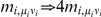, and thus it is a special case in the present formulation.

The No-Constraint model, in which there is no selection pressure on amino acid replacements (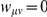), is a nucleotide substitution model extended to allow multiple nucleotide changes in infinitesimal time. Also, it is useful to note that the present model in the special case of 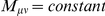 becomes equivalent to an amino acid substitution model converted into a codon substitution model; if 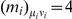 and 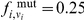, then 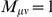 and Eq. 6 will become 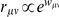 and equivalent to Eq. 4 with 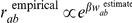.

In the present analyses, we assume for simplicity that 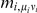 and 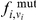 do not depend on codon position 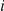; that is, 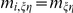 and 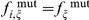, where 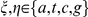. This approximation is reasonable because mutational tendencies may be independent of a nucleotide position in a codon. Let us define 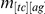 to represent the average of the exchangeabilities of the transversion type, 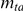, 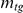, 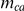, and 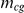, and likewise 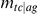 to represent the average of the exchangeabilities of the transition type, 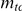 and 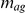. We use the ratios 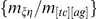 as parameters for exchangeabilities, and 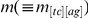 to represent the ratio of the exchangeability of double nucleotide change to that of single nucleotide change and also the ratio of the exchangeability of triple nucleotide change to that of double nucleotide change; note that the exchangeabilities of single, double, and triple nucleotide changes are of 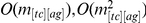, and 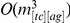 in Eq. 3, respectively, and that Eq. 3 must be satisfied. Then, multiple nucleotide changes in infinitesimal time can be completely neglected by making the parameter 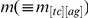 approach zero with keeping 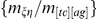 constant in Eq. 3. Also, it is noted that unlike the SDT model [20] double nucleotide changes at the first and the third positions in a codon are assumed to occur as frequently as doublet changes.

The number of parameters except equilibrium codon frequencies in the mechanistic codon substitution model is equal to 11; they are 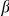, 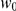, 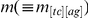, 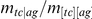, 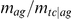, 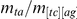, 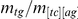, 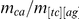, 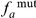, 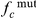, and 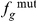, and fixed at certain values or optimized as ML parameters.

### Variations of mutation rate and of selective constraint across codon sites

Taking account of the variation of amino acid substitution rate over sites always increases the maximum likelihood of a phylogenetic tree in the analysis of amino acid sequences [31]. The variation of amino acid substitution rate can be caused by the variation of mutation rate and also by the variation of selective constraint on amino acids. Here, the variation of either mutation rate or selective constraint over sites is taken into account, but both are not taken into account at the same time because of a heavy computational load.

The variation of mutation rate over codon sites is also assumed to obey a 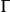 distribution [31] with a shape parameter 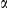 and the mean equal to 1, which is then approximated by a discrete-gamma distribution [32], [40] with 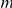 categories, each with equal probability, This model is specified with a suffix dG*m*r whose 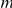 means the number of categories.

The variation of selective constraint over amino acid sites is assumed to obey a discrete-gamma distribution, too. In this model, the average of selective constraints over amino acid pairs (the mean acceptance rate), 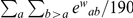 in the mechanistic codon substitution model or 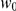 in other codon substitution models, is assumed to vary according to a discrete-gamma distribution. The rate matrix of each category is scaled so that the mean rate matrix satisfies Eq. 3. This model is specified with a suffix dG*m*s whose 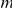 means the number of categories.

In the mechanistic codon substitution model, selective constraint 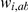 for 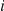
th category in a discrete-gamma distribution is calculated to satisfy the following equations.
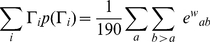
(14)

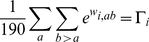
(15)

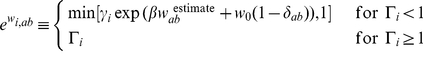
(16)where 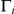 is the value of the 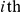 category in the discrete-gamma distribution whose mean is equal to the average of 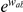 over all amino acid pairs and whose shape parameter is equal to 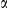; 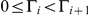. If 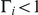 and 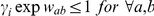, 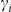 will be simply equal to a point of the discrete-gamma distribution whose mean is equal to 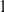.

In the other codon models, the equal amino acid constraint 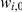 for 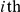 category in Eq. 4 and Eq. 5 is calculated from the following equations.
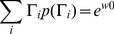
(17)

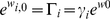
(18)In this case, 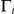 is and 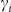 are points of the discrete-gamma distributions, whose means are equal to 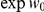 and 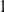, respectively, with the shape parameter 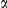.

The shape parameter 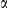 of the discrete-gamma distribution for the variation of mutation rate or selective constraint is optimized as one of ML parameters. Equal probability of each category is used for the mutation rate variation, but it may be inappropriate for the variation of selective constraint, because 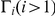 is often too small for a rate matrix to be significantly different between 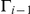 and 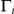. In such a case, the prior probability of 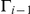 is increased to make the rate matrices for 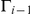 and 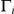 significantly different.

### A simple approximation for the variation of mutation rate over time

A mutation rate at each site may vary in each branch, especially long branches, of a phylogenetic tree. If the variation of mutation rate is synchronized among sites, it will be reflected by the length of each branch. The unsynchronized portion of rate variation among sites is considered. Here, a simple approximation for the variation of mutation rate over time is provided. The mutation rate matrix 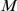 and therefore the substitution rate matrix 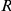 are assumed to vary in time only by a scalar factor, 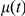 at time 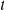. The expected values of the mean and the variance of the total substitution rate in a branch whose length is equal to 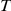 are as follows.
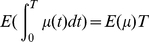
(19)

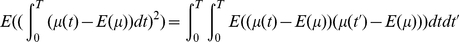


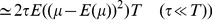
(20)The mutation rate as a function of time is assumed to be autocorrelated with a correlation time 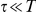. In this case, the mean and the variance are both the linear functions of 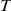. For the variation of the total mutation rate in the branch of the length 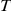, we assume a 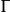 distribution whose scale and shape parameters are equal to 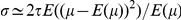 and 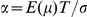, respectively. Then, the expected substitution matrix is:
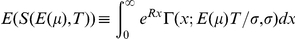


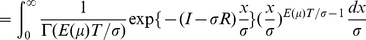


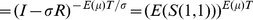
(21)where 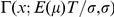 is the probability density function of a 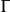 distribution with a scale parameter 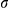 and a shape parameter equal to 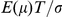, 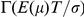 is the 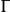 function, and 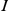 is the identity matrix. Then, 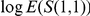 is used instead of 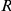 as a rate matrix; the rate matrix 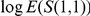 is scaled to make the mean rate matrix satisfy Eq. 3. A constant mutation rate corresponds to 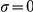. The scale parameter 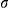 is set to 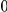 or is optimized as a ML parameter.

This approximation for the variation of mutation rate over time is very simple and does not require any additional computational time, although the performance will be limited in comparison with a more complete approximation [36]. However, the ML estimate of 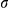 in this approximation may be influenced by the variation of mutation rate across sites, because the mean of the substitution matrix over sites is represented by a similar functional form to 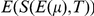; assuming that mutation rates vary across sites with a 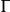 distribution, the mean of substitution matrix over sites for a branch of the length 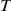 is formulated as 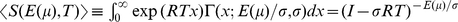, which is equal to the expected substitution matrix in the case of 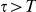.

### Datasets of protein-coding sequences used to evaluate codon substitution models

Substitution models are evaluated by using five datasets of codon sequences; (1) divergent and (2) closely-related chloroplast-encoded genes, (3) fast-evolving interspecific and (4) highly-polymorphic intraspecific mitochondrial genes, and (5) slowly-evolving nuclear genes.

1. Dataset cpDNA-9: Divergent codon sequences consisting of 45 protein-coding genes from 9 chloroplast genomes, whose protein sequences were used to estimate the cpREV10 by [8]; *Synechocystis PCC6803*, which was the outgroup sequence in their analysis, is not used in the present analysis. The codon sequences were obtained from the NCBI RefSeq database of organelle genomes. The total codon length of aligned genes is equal to 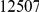, and the minimum amino acid identity between sequences is equal to 0.58. The tree topology that was estimated as Tree-1 by [8] is used here as the most probable tree. Overlapped segments between genes were removed from codon sequences.
2. Dataset cpDNA-55: Codon sequences consisting of 52 protein-coding genes from 55 chloroplast genomes of the major angiosperm lineages, which are genome sequences available in the NCBI RefSeq database out of the 64 genomes analyzed in [41], and which are genes owned by all 55 taxa. The tree topology estimated by [41] is used as the most probable tree in the present analysis. The total codon length of aligned genes is equal to 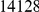, and the minimum amino acid identity between the sequences is equal to 0.73. The cpREV64 [38] was estimated from the full set of 77 protein-coding genes in the 64 genomes.
3. Dataset mammalian-mtDNA: Interspecific mammalian mitochondrial codon sequences consisting of 12 protein-coding genes from 69 mammalian species [42], whose genome sequences were obtained from the NCBI RefSeq database of organelle genomes. The total codon length of aligned genes is equal to 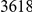, and the minimum amino acid identity between the sequences is equal to 0.66. The tree topology that was estimated as Tree-6 by [42] is used here as the most probable tree. Overlapped segments between genes were removed from codon sequences.
4. Dataset human-mtDNA: Intraspecific human mitochondrial codon sequences consisting of 12 protein-coding genes from 53 human races [43], whose genome sequences were obtained from a human mitochondrial genome database (MITOMAP). The total codon length of aligned genes is equal to 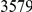, and the minimum amino acid identity between the sequences is equal to 0.99. The present analyses are done using the neighbor-joining tree topology estimated by [43]. Overlapped segments between genes were removed from codon sequences.
5. Dataset nDNA: Codon sequences of the 10 most slowly-evolving genes out of the 2789 nuclear genes of 10 mammals that were analyzed by [44]. The tree topologies estimated by [44] are used for respective genes and the tree-1 named by them is used here for the analyses of the concatenated genes. The total codon length of aligned genes is equal to 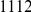, and the minimum amino acid identity between the sequences is equal to 0.97.

Homologous codon sequences are aligned every gene by ClustalW2 [45] that is modified to align codon sequences with codon score matrices [19]. The ML values for each model are calculated for each gene and also for the concatenated sequences of all genes by Phyml [46] also modified to analyze codon sequences.

### Statistical comparison of codon substitution models

Model selection must be pursued with considerable attention [47]. For the comparison of models one of which is a special case of the other, the likelihood ratio test (LRT) [48] can be used to test the superiority of a nesting model to nested models. Models that are not nesting or nested can be compared using Akaike information criterion (AIC) [49], Bayesian information criterion (BIC) [50], a decision-theoretical approach [51], [52], and the Bayes factor [53]. Here, AIC and BIC for a given tree topology of aligned codon sequences are used to compare codon substitution models derived from various empirical amino acid and codon substitution rate matrices and mechanistic codon substitution models with the wide range of selective constraint matrices. The AIC and BIC are defined as follows [18]:
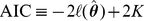
(22)

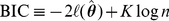
(23)where 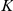 is the number of adjustable parameters, 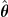 is the vector of the ML estimates of the parameters, 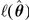 is the maximum log-likelihood value, and 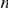 is the number of codons in a codon alignment. The model whose AIC or BIC is the minimum is regarded as the best model.

## Results and Discussion

The naming convention of the present models is briefly described in Table 1. In all models, the equilibrium frequencies of codons are estimated to be equal to codon frequencies in sequences. Other parameters including the scale parameter 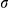 of a 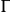 distribution for the variation of mutation rate over time are set to a certain value or optimized by maximizing the likelihood of a given topology of a phylogenetic tree. For the empirical amino acid substitution models converted into the codon substitution models, 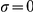 was assumed, because it seems not to be well matched with these models. In the empirical codon substitution model, 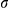 was optimized as well as 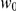. In the mechanistic codon substitution models, all 12 parameters including 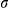 for the substitution rate matrix will be optimized if the AIC and the BIC values of a phylogenetic tree are decreased. For the separating analyses of human-mtDNA and the concatenating and the separating analyses of nDNA, which are both datasets consisting of highly-homologous sequences, the five parameters of 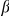, 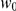, 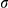, 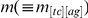, and 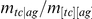 were optimized with 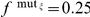 and 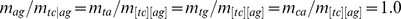. In all models, the variation of mutation rate or the variation of selective constraint over sites is taken into account. Both the variations over sites were approximated by a discrete-gamma distribution [32] with 4 categories. The shape parameter 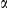 of the discrete-gamma distribution is optimized by maximizing the likelihood. Equal probability was used for each category in all models of rate variation. In the models of variable selective constraints, equal probability was used only for the non-mechanistic codon models for the cpDNA-9, and the different sets of prior probabilities on the basis of the values of 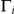 were used for the other models; 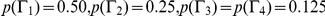 for the datasets cpDNA-9, cpDNA-55, and mammalian-mtDNA, and 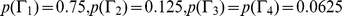 for the human-mtDNA and the nDNA. The AIC and the BIC are used for statistical comparisons of models.

**Table 1:** Brief description of models.

A. Empirical amino acid substitution models converted into codon substitution models	
JTT-*n* -F-dG*m*[rs],^a^ WAG-*n* -F-dG*m*[rs], LG-*n* -F-dG*m*[rs], cpREV10-*n* -F-dG*m*[rs], cpREV64-*n* -F-dG*m*[rs], mtREV-*n* -F-dG*m*[rs]	The empirical amino acid exchangeabilities of JTT [5], WAG [10], LG [11], cpREV10 [8], cpREV64 [38], and mtREV [6] are used as 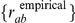 in Eq. 4. The suffix *n* means the number of parameters optimized for the substitution rate matrix; the 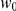 is a ML parameter when 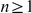 .

a In the models specified with the suffix “F”, equilibrium codon frequencies are assumed to be equal to codon frequencies in codon sequences. dG*m*[rs], i.e., dG*m*r or dG*m*s, means that the variation of mutation rate or selective constraint over site is approximated by a discrete gamma distribution with *m* categories [32], respectively; 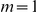 means no variation and the suffix dG*1*[rs] is omitted.

### Mechanistic codon substitution models outperform other substitution models

First, each gene in a dataset is separately aligned and then all aligned sequences are concatenated. The maximum log-likelihood values of a given phylogenetic tree of concatenated genes for various codon substitution models are listed in Tables 2, 3, 4, 5, 6 for cpDNA-9, cpDNA-55, mammalian-mtDNA, human-mtDNA, and nDNA, respectively. Values in parentheses indicate that the corresponding parameters are fixed at the value specified. The maximum log-likelihood (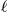), AIC and BIC values for each model are listed in these tables with the difference (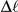, 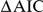, and 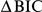) from those of a reference model. For the datasets cpDNA-9, cpDNA-55, and nDNA that use the universal codon table, the empirical codon substitution model KHG-2-F-dG4s estimated from nuclear-encoded sequences is used as a reference state; in the KHG-2-F-dG4s, 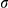 is optimized as well as 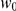. For mitochondrial genomes that use a minor genetic code, no empirical codon substitution rate matrix is available, and so the codon substitution model, mtREV-1-dG4s, which is converted from the empirical amino acid substitution matrix mtREV estimated from mitochondrial proteins, is used as a reference state; in the mtREV-1-F-dG4s, 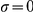 is assumed, and only 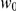 is optimized.

**Table 2:** Comparisons between various codon substitution models in the concatenating analysis of cpDNA-9.

Codon substitution model^a^	K^b^	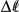 c	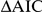 c	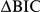 c	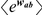 d e	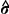 e f	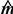 e g	h	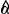 i
cpREV64-1-F-dG4r	62	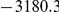	6358.6	6351.2		(0.0)			0.977
LG-1-F-dG4r	62	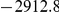	5823.6	5816.1		(0.0)			0.973
JTT-1-F-dG4r	62	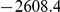	5214.8	5207.4		(0.0)			1.020
WAG-1-F-dG4r	62	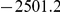	5000.5	4993.0		(0.0)			1.148
cpREV10-1-F-dG4r	62	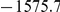	3149.3	3141.9		(0.0)			1.195
cpREV64-1-F-dG4s	62	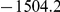	3006.4	2999.0		(0.0)			0.505
LG-1-F-dG4s	62	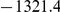	2640.8	2633.4		(0.0)			0.496
WAG-1-F-dG4s	62	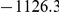	2250.5	2243.1		(0.0)			0.573
JTT-1-F-dG4s	62	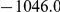	2090.0	2082.6		(0.0)			0.519
cpREV10-1-F-dG4s	62	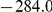	566.0	558.6		(0.0)			0.591
KHG-2-F-dG4r	63	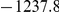	2475.7	2475.7		0.031			1.301
KHG-2-F-dG4s	63	0.0	0.0	0.0		0.290			0.575
No-Constraint-10-F-dG4r	71	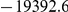	38801.2	38860.7	(1.0)	0.000	0.040	2.541	1.830
Equal-Constraint-11-F-dG4r	72	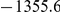	2729.2	2796.1	0.021	0.424	0.292	2.053	1.178
EI-12-F-dG4r	73	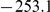	526.3	600.6	0.023	0.000	0.494	2.217	1.160
JTT-ML91+-12-F-dG4r	73	288.7	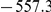	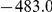	0.018	0.002	0.569	1.702	1.131
WAG-ML91+-12-F-dG4r	73	477.4	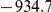	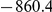	0.015	0.272	0.526	2.184	1.126
KHG-ML200-12-F-dG4r	73	562.9	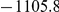	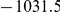	0.039	0.000	0.325	1.610	1.122
LG-ML91+-12-F-dG4r	73	627.3	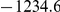	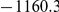	0.023	0.000	0.485	2.158	1.144
Equal-Constraint-11-F-dG4s	72	680.2	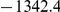	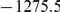	0.063	0.414	0.208	2.196	0.384
EI-12-F-dG4s	73	1935.2	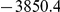	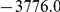	0.060	0.000	0.431	2.307	0.390
JTT-ML91+-12-F-dG4s	73	2640.2	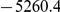	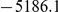	0.052	0.125	0.461	1.774	0.363
KHG-ML200-12-F-dG4s	73	2646.5	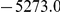	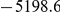	0.106	0.170	0.215	1.705	0.388
WAG-ML91+-12-F-dG4s	73	2827.2	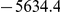	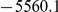	0.048	0.313	0.405	2.349	0.359
LG-ML91+-12-F-dG4s	73	2956.6	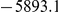	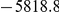	0.064	0.201	0.364	2.369	0.370
LG-ML91+-11s-F-dG4s	72	2412.8	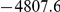	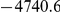	0.066	2.335	(0.0)	2.667	0.297
LG-ML91+-11-F-dG4s	72	2942.4	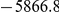	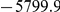	0.066	(0.0)	0.409	2.292	0.385
LG-ML91+-12-F	72	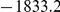	3684.4	3751.4	0.026	0.878	0.622	2.039	

a The prior probability of each category for the mechanistic codon models of “dG4s” is 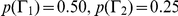, and 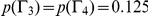; equal probability is used in other models.

b The number of adjustable parameters.

c Differences from the reference state; 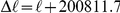, 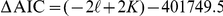, and 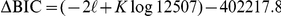.

d The average of 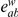 over all amino acid pairs 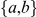; 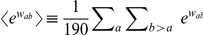.

e The value parenthesized means that the parameter is fixed at the value specified.

f The scale parameter of a 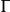 distribution for the variation of mutation rate over time.

g The ratio of double to single and of triple to double nucleotide change exchangeability; 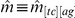.

h The ratio of mean transitional to mean transversional exchangeability; 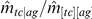.

i The shape parameter of a discrete gamma distribution for the variation of mutation rate or selective constraint over sites.

**Table 3:** Comparisons between various codon substitution models in the concatenating analysis of cpDNA-55.

Codon substitution model^a^	K^b^	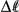 c	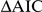 c	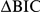 c	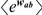 d e	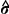 e f	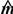 e g	h	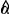 i
LG-1-F-dG4r	62	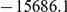	31370.2	31362.6		(0.0)			1.055
WAG-1-F-dG4r	62	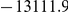	26221.8	26214.3		(0.0)			1.094
cpREV10-1-F-dG4r	62	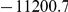	22399.4	22391.8		(0.0)			1.096
JTT-1-F-dG4r	62	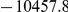	20913.6	20906.1		(0.0)			1.092
cpREV64-1-F-dG4r	62	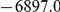	13792.0	13784.5		(0.0)			1.091
LG-1-F-dG4s	62	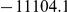	22206.2	22198.7		(0.0)			0.289
WAG-1-F-dG4s	62	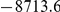	17425.2	17417.7		(0.0)			0.300
cpREV10-1-F-dG4s	62	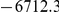	13422.7	13415.1		(0.0)			0.298
JTT-1-F-dG4s	62	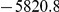	11639.7	11632.1		(0.0)			0.299
cpREV64-1-F-dG4s	62	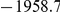	3915.4	3907.8		(0.0)			0.299
KHG-2-F-dG4r	63	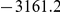	6322.5	6322.5		0.068			1.073
KHG-2-F-dG4s	63	0.0	0.0	0.0		0.150			0.277
No-Constraint-10-F-dG4r	71	1705.9	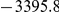	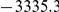	(1.0)	0.000	0.018	3.557	1.055
Equal-Constraint-11-F-dG4r	72	26281.9	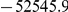	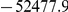	0.156	0.000	0.101	2.671	1.107
EI-12-F-dG4r	73	26941.5	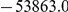	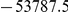	0.143	0.000	0.107	2.732	1.100
JTT-ML91+-12-F-dG4r	73	27198.5	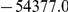	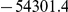	0.122	0.000	0.122	2.501	1.111
WAG-ML91+-12-F-dG4r	73	27378.4	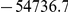	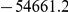	0.125	0.000	0.115	2.690	1.100
LG-ML91+-12-F-dG4r	73	27664.8	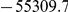	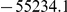	0.142	0.000	0.112	2.707	1.109
KHG-ML200-12-F-dG4r	73	27683.4	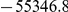	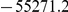	0.163	0.000	0.099	2.479	1.106
Equal-Constraint-11-F-dG4s	72	34659.7	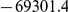	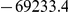	0.276	0.124	0.056	2.664	0.259
EI-12-F-dG4s	73	35716.3	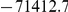	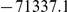	0.235	0.103	0.071	2.727	0.247
KHG-ML200-12-F-dG4s	73	36243.5	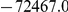	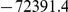	0.251	0.116	0.058	2.477	0.285
JTT-ML91+-12-F-dG4s	73	36257.9	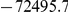	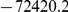	0.204	0.072	0.098	2.438	0.231
WAG-ML91+-12-F-dG4s	73	36362.6	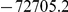	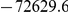	0.222	0.109	0.074	2.670	0.234
LG-ML91+-12-F-dG4s	73	36583.3	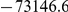	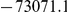	0.233	0.105	0.073	2.701	0.256
LG-ML91+-11s-F-dG4s	72	36336.9	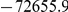	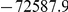	0.250	0.260	(0.0)	2.788	0.237
LG-ML91+-11-F-dG4s	72	36479.9	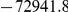	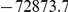	0.213	(0.0)	0.123	2.623	0.273
LG-ML91+-12-F	72	14390.7	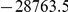	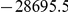	0.135	0.000	0.182	2.569	

a The prior probability of each category for the “dG4s” is 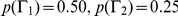, and 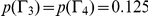.

b The number of adjustable parameters.

c Differences from the reference state; 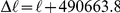, 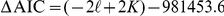, and 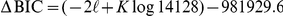.

d The average of 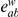 over all amino acid pairs 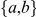; 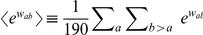.

e The value parenthesized means that the parameter is fixed at the value specified.

f The scale parameter of a 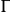 distribution for the variation of mutation rate over time.

g The ratio of double to single and of triple to double nucleotide change exchangeability; 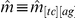.

h The ratio of mean transitional to mean transversional exchangeability; 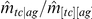.

i The shape parameter of a discrete gamma distribution for the variation of mutation rate or selective constraint over sites.

**Table 4:** Comparisons between various codon substitution models in the concatenating analysis of mammalian-mtDNA.

Codon substitution model^a^	K^b^	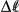 c	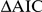 c	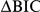 c	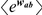 d e	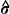 e f	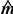 e g	h	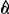 i
WAG-1-F-dG4r	62	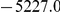	10454.0	10454.0		(0.0)			0.786
LG-1-F-dG4r	62	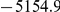	10309.8	10309.8		(0.0)			0.771
JTT-1-F-dG4r	62	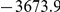	7347.7	7347.7		(0.0)			0.783
mtREV-1-F-dG4r	62	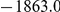	3725.9	3725.9		(0.0)			0.870
WAG-1-F-dG4s	62	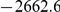	5325.2	5325.2		(0.0)			0.327
LG-1-F-dG4s	62	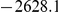	5256.1	5256.1		(0.0)			0.314
JTT-1-F-dG4s	62	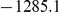	2570.2	2570.2		(0.0)			0.329
mtREV-1-F-dG4s	62	0.0	0.0	0.0		(0.0)			0.339
No-Constraint-10-F-dG4r	71	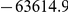	127247.9	127303.6	(1.0)	0.000	0.000	4.908	1.965
Equal-Constraint-11-F-dG4r	72	464.7	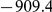	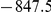	0.013	0.000	0.108	4.508	0.495
EI-12-F-dG4r	73	4336.4	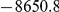	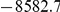	0.007	0.000	0.271	4.697	0.928
KHG-ML200-12-F-dG4r	73	5340.3	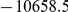	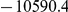	0.022	0.000	0.088	3.238	0.480
JTT-ML91+-12-F-dG4r	73	5501.7	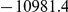	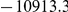	0.006	0.000	0.228	3.679	0.452
WAG-ML91+-12-F-dG4r	73	5728.6	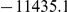	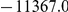	0.006	0.000	0.206	5.614	0.492
LG-ML91+-12-F-dG4r	73	6315.1	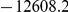	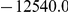	0.009	0.000	0.147	5.921	0.515
Equal-Constraint-11-F-dG4s	72	6961.9	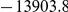	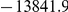	0.036	1.313	0.031	4.984	0.269
EI-12-F-dG4s	73	10402.4	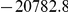	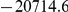	0.024	1.137	0.124	5.426	0.267
KHG-ML200-12-F-dG4s	73	11621.0	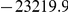	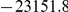	0.063	1.119	0.039	3.658	0.306
JTT-ML91+-12-F-dG4s	73	11698.3	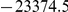	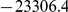	0.022	1.637	0.091	4.189	0.259
WAG-ML91+-12-F-dG4s	73	11997.4	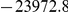	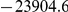	0.020	1.686	0.092	6.588	0.259
LG-ML91+-12-F-dG4s	73	12532.5	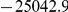	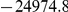	0.028	1.826	0.065	7.158	0.262
LG-ML91+-11-F-dG4s	72	12113.1	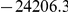	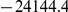	0.035	(0.0)	0.128	6.009	0.290
LG-ML91+-11s-F-dG4s	72	12268.3	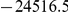	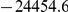	0.028	3.066	(0.0)	7.600	0.252
LG-ML91+-12-F	72	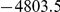	9627.1	9689.0	0.011	3.713	0.196	5.477	

a The prior probability of each category for the “dG4s” is 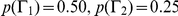, and 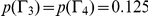.

b The number of adjustable parameters.

c Differences from the reference state; 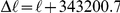, 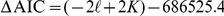, and 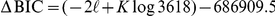.

d The average of 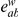 over all amino acid pairs 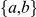; 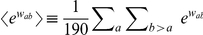.

e The value parenthesized means that the parameter is fixed at the value specified.

f The scale parameter of a 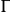 distribution for the variation of mutation rate over time.

g The ratio of double to single and of triple to double nucleotide change exchangeability; 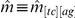.

h The ratio of mean transitional to mean transversional exchangeability; 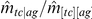.

i The shape parameter of a discrete gamma distribution for the variation of mutation rate or selective constraint over sites.

**Table 5:** Comparisons between various codon substitution models in the concatenating analysis of human-mtDNA.

Codon substitution model^a^	K^b^	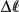 c	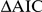 c	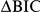 c	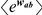 d e	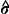 e f	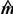 e g	h	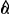 i
LG-1-F-dG4r	62	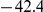	84.8	84.8		(0.0)			0.338
WAG-1-F-dG4r	62	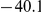	80.1	80.1		(0.0)			0.343
JTT-1-F-dG4r	62	5.9	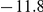	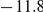		(0.0)			0.345
mtREV-1-F-dG4r	62	7.7	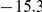	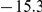		(0.0)			0.331
LG-1-F-dG4s	62	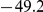	98.3	98.3		(0.0)			0.080
WAG-1-F-dG4s	62	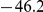	92.5	92.5		(0.0)			0.079
JTT-1-F-dG4s	62	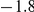	3.7	3.7		(0.0)			0.091
mtREV-1-F-dG4s	62	0.0	0.0	0.0		(0.0)			0.085
No-Constraint-10-F-dG4r	71	315.1	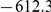	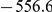	(1.0)	0.000	0.000	47.760	0.465
Equal-Constraint-11-F-dG4r	72	515.6	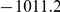	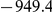	0.092	0.066	0.000	39.685	0.612
EI-12-F-dG4r	73	525.8	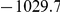	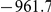	0.076	0.000	0.000	36.208	0.620
KHG-ML200-12-F-dG4r	73	530.8	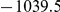	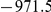	0.110	0.000	0.000	31.182	0.648
WAG-ML91+-12-F-dG4r	73	535.5	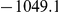	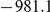	0.069	0.000	0.000	41.404	0.635
LG-ML91+-12-F-dG4r	73	535.7	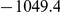	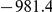	0.089	0.002	0.000	42.787	0.627
JTT-ML91+-12-F-dG4r	73	541.0	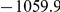	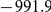	0.051	0.000	0.000	32.733	0.646
Equal-Constraint-11-F-dG4s	72	517.4	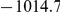	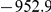	0.108	0.000	0.000	36.267	0.106
EI-12-F-dG4s	73	528.3	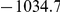	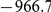	0.079	0.000	0.000	34.994	0.123
KHG-ML200-12-F-dG4s	73	536.6	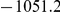	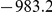	0.106	0.000	0.000	30.457	0.227
LG-ML91+-12-F-dG4s	73	538.4	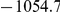	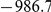	0.078	0.000	0.000	39.024	0.233
WAG-ML91+-12-F-dG4s	73	539.3	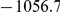	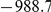	0.059	0.000	0.000	38.794	0.207
JTT-ML91+-12-F-dG4s	73	542.6	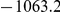	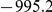	0.049	0.000	0.000	32.064	0.168
JTT-ML91+-11-F-dG4s	72	542.6	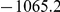	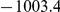	0.049	(0.0)	0.000	32.067	0.168
JTT-ML91+-11s-F-dG4s	72	542.6	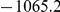	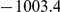	0.049	0.000	(0.0)	32.067	0.168
JTT-ML91+-12-F	72	522.3	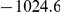	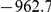	0.052	0.000	0.000	32.207	

a The prior probability of each category for the “dG4s” is 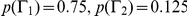, and 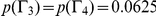.

b The number of adjustable parameters.

c Differences from the reference state; 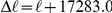, 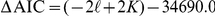, and 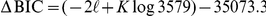.

d The average of 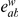 over all amino acid pairs 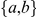; 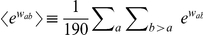.

e The value parenthesized means that the parameter is fixed at the value specified.

f The scale parameter of a 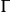 distribution for the variation of mutation rate over time.

g The ratio of double to single and of triple to double nucleotide change exchangeability; 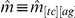.

h The ratio of mean transitional to mean transversional exchangeability; 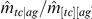.

i The shape parameter of a discrete gamma distribution for the variation of mutation rate or selective constraint over sites.

**Table 6:** Comparisons between various codon substitution models in the concatenating analysis of nDNA.

Codon substitution model^a^	K^b^	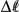 c	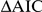 c	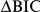 c	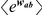 d e	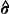 e f	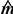 e g	h	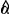 i
LG-1-F-dG4r	62	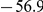	111.7	106.7		(0.0)			0.429
WAG-1-F-dG4r	62	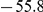	109.6	104.6		(0.0)			0.431
JTT-1-F-dG4r	62	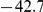	83.3	78.3		(0.0)			0.434
LG-1-F-dG4s	62	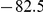	163.1	158.1		(0.0)			0.102
WAG-1-F-dG4s	62	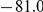	160.1	155.0		(0.0)			0.103
JTT-1-F-dG4s	62	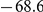	135.3	130.3		(0.0)			0.110
KHG-2-F-dG4r	63	20.5	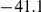	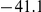		0.082			0.472
KHG-2-F-dG4s	63	0.0	0.0	0.0		0.214			0.118
No-Constraint-3-F-dG4r	64	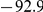	187.9	192.9	(1.0)	0.000	0.000	4.368	0.499
Equal-Constraint-4-F-dG4r	65	137.1	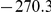	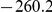	0.083	0.000	0.020	2.638	0.465
KHG-ML200-5-F-dG4r	66	143.2	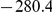	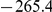	0.085	0.000	0.018	2.500	0.461
EI-5-F-dG4r	66	144.6	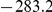	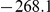	0.061	0.000	0.026	2.675	0.458
LG-ML91+-5-F-dG4r	66	146.6	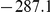	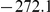	0.065	0.000	0.025	2.756	0.456
JTT-ML91+-5-F-dG4r	66	147.3	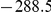	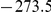	0.050	0.000	0.027	2.515	0.463
WAG-ML91+-5-F-dG4r	66	147.7	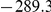	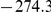	0.053	0.000	0.025	2.737	0.459
Equal-Constraint-4-F-dG4s	65	119.4	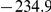	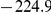	0.092	0.003	0.032	2.470	0.109
KHG-ML200-5-F-dG4s	66	124.4	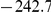	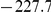	0.080	0.000	0.039	2.333	0.157
EI-5-F-dG4s	66	125.9	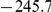	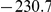	0.060	0.000	0.049	2.450	0.152
LG-ML91+-5-F-dG4s	66	125.9	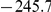	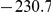	0.063	0.000	0.050	2.466	0.172
JTT-ML91+-5-F-dG4s	66	128.0	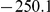	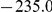	0.051	0.000	0.053	2.344	0.133
WAG-ML91+-5-F-dG4s	66	128.4	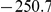	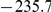	0.056	0.000	0.049	2.503	0.127
WAG-ML91+-4s-F-dG4r	65	146.1	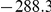	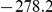	0.055	0.016	(0.0)	2.755	0.449
WAG-ML91+-4-F-dG4r	65	147.7	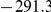	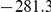	0.053	(0.0)	0.025	2.737	0.459
WAG-ML91+-4s-F-dG4s	65	127.5	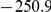	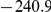	0.057	0.079	(0.0)	2.572	0.133
WAG-ML91+-4-F-dG4s	65	128.4	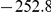	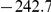	0.056	(0.0)	0.049	2.507	0.129
WAG-ML91+-5-F	65	109.7	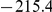	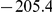	0.055	0.001	0.059	2.535	

a In the models specified with the suffix “-3-”, “-4-”, “-4s-” or “-5-”, three, four or five parameters are optimized with 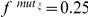 and 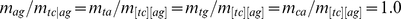. The prior probability of each category for the “dG4s” is 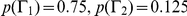, and 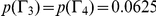.

b The number of adjustable parameters.

c Differences from the reference state; 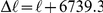, 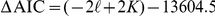, and 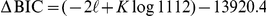.

d The average of 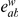 over all amino acid pairs 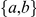; 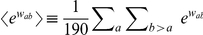.

e The value parenthesized means that the parameter is fixed at the value specified.

f The scale parameter of a 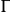 distribution for the variation of mutation rate over time.

g The ratio of double to single and of triple to double nucleotide change exchangeability; 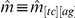.

h The ratio of mean transitional to mean transversional exchangeability; 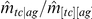.

i The shape parameter of a discrete gamma distribution for the variation of mutation rate or selective constraint over sites.

In the case of mitochondrial genes, i.e., mammalian-mtDNA and human-mtDNA, the models based on mtREV always show the smallest 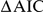 and 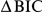, i.e., the best performance, in the empirical amino acid substitution models converted into the codon substitution models. For the dataset cpDNA-55, the models converted from cpREV64 show the best performance in the models converted from the empirical amino acid substitution models, and the models converted from cpREV10 perform best for the dataset cpDNA-9. These results are reasonable because the amino acid substitution probability matrix mtREV [6] was estimated from mitochondrial proteins, and cpREV64 [38] and cpREV10 [8] were estimated from the full sets of chloroplast proteins corresponding to cpDNA-55 and cpDNA-9, respectively; see the method section. A rather interesting result is that the models converted from cpREV64 shows larger 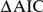 and 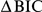 for cpDNA-9 than the models converted from LG, WAG, and JTT that were estimated from nuclear-encoded proteins, This fact indicates that substitution tendencies vary between genes and cannot always be represented by the average tendencies of substitutions. Delport et al.[16] showed that the empirical substitution matrices represent the average tendencies of substitutions over various protein families by sacrificing gene-level resolution.

The empirical codon substitution model KHG performs significantly better for chloroplast-encoded and nuclear-encoded genes than all the amino acid substitution models converted into the codon models. It has often be insisted that synonymous substitutions are saturated between distantly related genes and so substitution analyses at the codon level hardly include more information than those at the amino acid level. However, a fact that KHG performs better even for the distantly related sequence family (cpDNA-9) than the models converted from cpREV10 indicates that codon sequences include more information than amino acid sequences even in the case of distantly related sequences.

If the amino acid substitution models converted into codon models are compared with the mechanistic codon substitution models, the superiority of the codon substitution models will be clearer. For all datasets, the mechanistic codon models with the various estimates of selective constraints show significantly lower 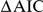 and 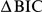 than the amino acid substitution models converted into the codon models. The Equal-Constraint model always performs worst, and is far inferior to the amino acid dependent constraint models for the phylogenetic trees including long branches such as the datasets cpDNA-9 and mammalian-mtDNA. Only for the phylogenetic trees consisting of extremely short branches such as the datasets human-mtDNA and nDNA, it is not remarkably worse than the amino acid dependent constraint models; amino acid identities between sequences are equal to or larger than 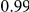 in human-mtDNA and 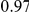 in nDNA. Consistently, 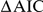 and 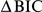 for the No-Constraint model, which is essentially equivalent to a nucleotide substitution model, are extremely larger for cpDNA-9 and mammalian-mtDNA, but smaller for cpDNA-55 and human-mtDNA than those for the reference model. These results can be explained to be because the amino acid dependencies of selective constraints must be taken into account to correctly evaluate amino acid substitutions, which occur in long branches, in order to precisely estimate branch lengths. One of the interesting facts is that the No-Constraint model is better for cpDNA-55 and human-mtDNA but worse for nDNA than the reference model, even though the phylogenetic tree of nDNA consists of short branches. This characteristic feature results from a fact that the genes in nDNA are slowly-evolving genes with strong selective constraints on amino acids; note that sequences in the dataset nDNA are highly homologous with amino acid identities greater than 0.97 but are collected from a wide range of mammalian species, i.e., *Borentheria*, *Xenarthra*, and *Afrotheria*.

The EI model, in which the selective constraints were evaluated on the basis of average contact energies between residues in proteins [19], always show better performance than the Equal-Constraint model but is always inferior to the other models, which use the selective constraints estimated from the empirical amino acid substitution frequency matrices, especially for the datasets cpDNA-9 and mammalian-mtDNA including long branches. The similar result was obtained in [19]. The selective constraint matrix LG-ML91+ performs better on average than the WAG-ML91+, JTT-ML91+, and KHG-ML200, although the differences of 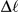 between them are small in comparison with the differences from the EI. An unexpected fact is that the selective constraint matrix KHG-ML200 estimated from the codon substitution rate matrix KHG tends to be inferior to the other selective constraint matrices estimated from the empirical amino acid substitution rate matrices, LG-ML91+, WAG-ML91+, and LG-ML91+, although it performs better except for nDNA than the EI.

**Figure 1:**
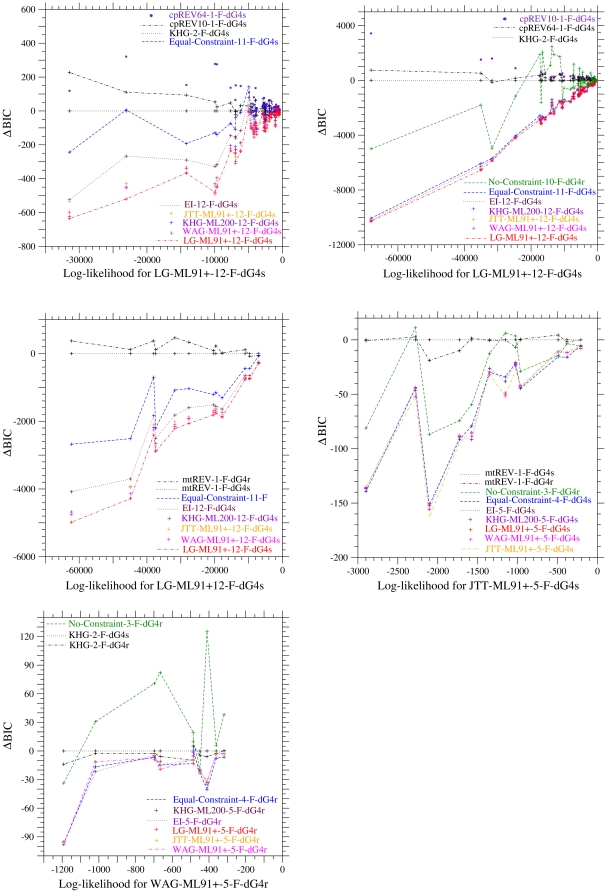
Comparisons of 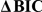 of each gene in each dataset among models. 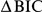
 of each gene in cpDNA-9 (A), cpDNA-55 (B), mammalian-mtDNA (C), human-mtDNA (D), and nDNA (E) for each specified model is plotted against its log-likelihood value for the best model in the concatenating analysis of the genes. The horizontal dotted line of 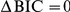 shows the reference model for each dataset. The best model is shown by the lowest dot-dashed line. The lower dotted line in each figure shows the data points for the EI model. The lower and the upper broken lines show the Equal-Constraint and the No-Constraint models, respectively. The No-Constraint model is not shown for cpDNA-9 and mammalian-mtDNA, because its 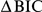 values are too large. In the models specified with the suffix “-5-” for human-mtDNA and nDNA, five parameters were optimized with 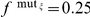 and 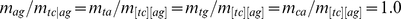.

In the concatenating analyses of multiple genes, it is assumed that all genes have no difference in equilibrium codon frequencies, nucleotide exchangeabilities, and the variations of mutation rate and of selective constraint. These assumptions are not always appropriate. Thus, the separating analyses of multiple genes have been carried out. The 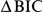 of each gene for some models are plotted against the maximum log-likelihood value for the best model in Fig. 1 for all datasets. In all datasets, the mechanistic codon substitution models show significantly lower 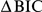 than the best amino acid substitution model converted into the codon models, for almost all genes except some genes for which the maximum log-likelihood values are large owing to short sequences. The No-Constraint model is not shown for cpDNA-9 and mammalian-mtDNA, because its 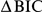 values for them are too large to show. For the phylogenetic trees of cpDNA-55 and human-mtDNA consisting of relatively short branches, the No-Constraint model, i.e., a nucleotide substitution model, is better for most of the genes than the amino acid substitution models converted into the codon models, as also indicated by the concatenating analyses. Even for those datasets, 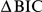 can be further decreased by the mechanistic codon substitution models including the the Equal-Constraint model. However, differences of 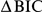 between the mechanistic substitution models with the different selective constraints are small for those dataset in comparison with the improvement from the amino acid substitution models converted into the codon models. For the phylogenetic trees of cpDNA-9 and mammalian-mtDNA consisting of long branches, the differences between the Equal-Constraint and the EI and between the EI and the best model with amino acid dependent selective constraints are very significant, as indicated by the concatenating analyses.

The mechanistic codon substitution model performs better for a wide range of sequences from highly-homologous to highly-diverged sequences than both nucleotide and amino acid substitution models. This is because it takes into account both mutational tendencies at the nucleotide level and selection at the amino acid level.

### Variable mutation rates versus variable selective constraints over sites

Significance of rate variation over sites in proteins has been demonstrated in nucleotide substitution models and amino acid substitution models [32], [33]. These results do not necessarily indicate the variation of mutation rate over sites, because the variation of selective constraint over sites in proteins can also cause the variation of amino acid substitution rate over sites even under a uniform mutation rate over sites. Here, we examine which model better fits the heterogeneity of amino acid substitution rate over sites.

**Figure 2:**
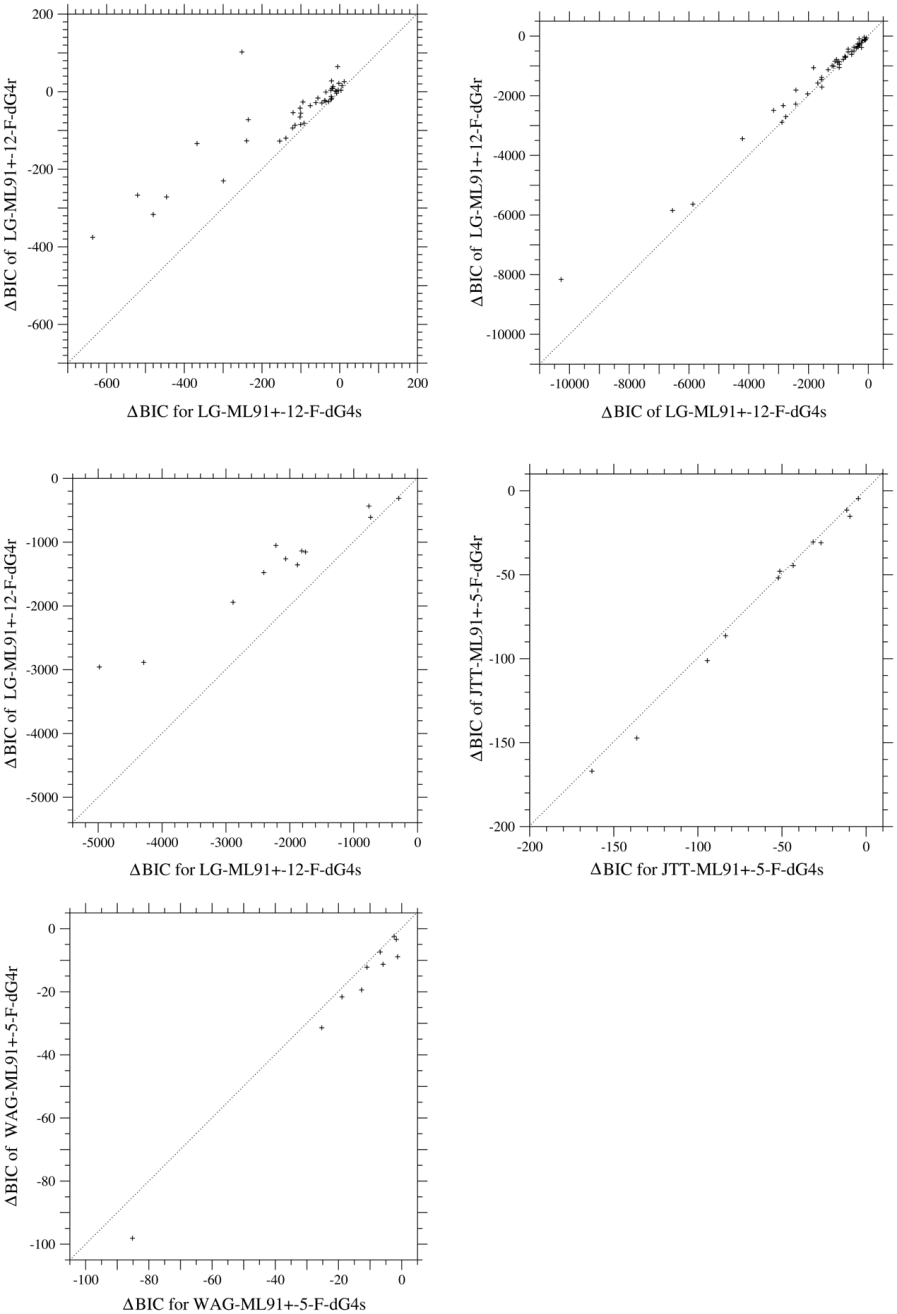
Comparison of 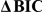 of each gene in each dataset between the models with variable mutation rates and with variable selective constraints over sites. 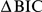
 of each gene in cpDNA-9 (A), cpDNA-55 (B), mammalian-mtDNA (C), human-mtDNA (D), and nDNA (E) is compared between the models with the variation of mutation rate (dG4r) and with the variation of selection constraint (dG4s) over sites. The dotted line shows the line of equal values between the ordinate and the abscissa. In the models specified with the suffix “-5-” for human-mtDNA, five parameters were optimized with 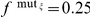 and 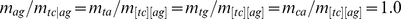.

The discrete gamma distribution with 4 categories has been used to emulate both the variations of selective constraint and of mutation rate over sites. The models with variable selective constraints and with variable mutation rates are specified by dG4s and dG4r, respectively. Tables 2, 3, 4 for the concatenating analyses of genes consistently indicate that the codon substitution models with the variation of selective constraint (dG4s) show significantly lower 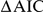 and 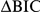 than the corresponding models with the variation of mutation rate (dG4r) over sites for the datasets cpDNA-9, cpDNA-55, and mammalian-mtDNA. The comparisons of 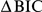 of each gene between those two types of the models are shown in Fig. 2 for all datasets. These figures also show that the variation of selective constraint is a statistically better model than the variation of mutation rate at least for cpDNA-9, cpDNA-55 and mammalian-mtDNA. This is reasonable because a mutation rate may not significantly differ among sites in a gene but selective constraints originating in the tertiary structure and the function of a protein should vary among sites in a protein. Generally speaking, selective constraints on amino acid replacements are stronger in a protein core than on protein surface [54].

However, in both the concatenating analyses and the separating analyses of genes, the 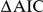 and the 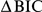 values for the models with the variation of selective constraint are not smaller for the nDNA than those for the models with the variation of mutation rate. For the human-mtDNA consisting of highly-polymorphic intraspecific mitochondrial genes, the mechanistic codon models with the variation of selective constraints attain slightly lower 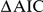 and 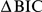 than the corresponding models with rate variation, although the differences of 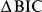 between the two models are insignificant in the separating analyses of the genes. The phylogenetic trees of the datasets human-mtDNA and nDNA consist of extremely short branches only, in which nonsynonymous substitutions insignificantly occur under strong selective constraints. In such a phylogenetic tree, it is hard to estimate correctly the variation of selective constraint over sites as indicated by the high performance of the Equal-Constraint model. This would be the reason why the differences of 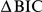 between the mechanistic codon models of the dG4r and the dG4s are insignificant in the separating analyses of genes for the human-mtDNA. On the other hand, the present result for the nDNA, which consists of 10 genes that are not necessarily closely-located in the same chromosome, may indicate the possibility of rate variation over sites.

### Site dependencies of selective constraints

Selective constraints against amino acid replacements at each site must reflect both structural and functional constraints on a residue type at each site, which are required for a protein to fold into a unique native structure and to properly function, and vary among residue sites in a protein. Here a simple analysis of site dependencies of selective constraints has been performed to ascertain the correlation between selective constraints and structural constrains at each site.

**Figure 3:**
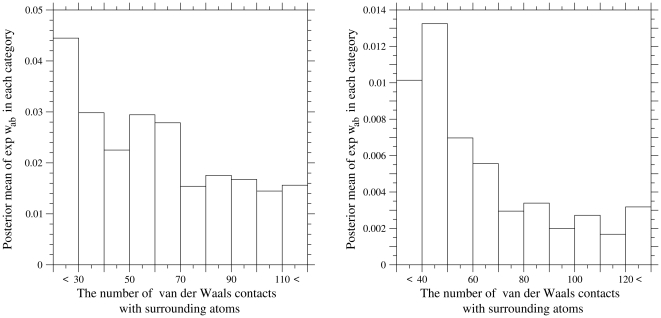
Site dependences of selective constraints. Site dependences of selective constraints in the photosystem II CP47 protein (psbB gene) (A) and cytochrome c oxidase subunit 1 mitochondrial protein (COX1 gene) (B) are shown. Residue sites are categorized by the number of van der Waals contacts with surrounding non-solvent atoms in the protein structure; neighboring residues along a polypeptide chain are not counted. The degree of van der Waals contact for an atom pair, which is separated by 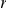 and whose van der Waals distance is equal to 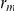, is defined as 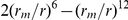 for 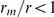 and 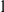 for 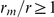. The van der Waals contacts are evaluated for the psbB in the 38-meric state of the photosystem II complex from *Thermosynechococcus vulcanus*, and for the COX1 in the biological 26-meric state of bovine heart cytochrome C oxidase in the fully reduced state; the protein coordinates 3ARC and 2EIJ in the PDB database were used. Posterior mean of selective constrains (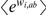) averaged over sites in each residue category is shown in the ordinate. The posterior mean of selective constrains were calculated by the LG-ML91+-12-F-dG4s for the concatenated sequences of the datasets cpDNA-9 and mammalian-mtDNA.

Site dependencies of selective constraints are evaluated [40] as a posterior mean of 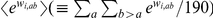 over categories 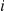 for each site. Residue sites are categorized by the number of van der Waals contacts with surrounding non-solvent atoms in a protein structure, which are supposed to reflect the strength of structural constraints; neighboring residues along a polypeptide chain are not counted. Then, the posterior mean of 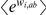 are averaged over sites in each residue category and its dependence on the category is examined. In Fig. 3, the site dependencies of selective constrains are shown for the photosystem II CP47 chloroplast protein (psbB gene) and for the cytochrome c oxidase subunit 1 mitochondrial protein (COX1 gene). The van der Waals contacts were evaluated for the psbB in the 38-meric state of the photosystem II protein complex and for the COX1 in the biological 26-meric state of bovine heart cytochrome C oxidase in the fully reduced state; the protein coordinates 3ARC and 2EIJ in the PDB database were used. The posterior mean of selective constrains for each site was calculated in the LG-ML91+-12-F-dG4s for the concatenated sequences of the datasets cpDNA-9 and mammalian-mtDNA. It is clear that the selective constraints tend to be stronger at residues surrounded by more atoms, indicating that they reflect structural constraints at each residue site in a protein. Here we have taken account of purifying selection only, but positive selection can be also examined [29] in terms of 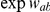 (fixation rate) at each site.

### Estimates of branch lengths under different models

The correct estimation of branch length is critical for the estimations of phylogeny and divergence times. It is known that branch-length estimation is significantly influenced by model selection. Yang et al. [33] found that branch lengths are severely underestimated by nucleotide substitution models in which rate variation over site is ignored. Also they found that simpler and worse models tend to underestimate branch lengths more severely, and such a bias is more serious for longer branches.

**Figure 4:**
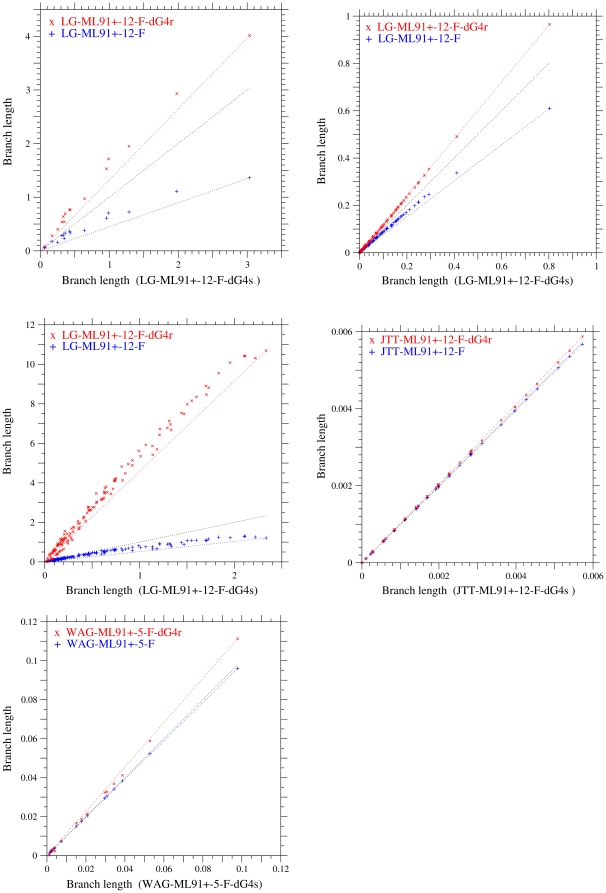
Comparisons of branch lengths estimated by the models with a uniform rate, variable mutation rates, and variable selective constraints over sites. Branch lengths estimated for the phylogenetic trees of cpDNA-9 (A), cpDNA-55 (B), mammalian-mtDNA (C), human-mtDNA (D), and nDNA (E) are compared among models. The abscissa shows the branch lengths estimated by the model with the variation of selection constraint (dG4s). The LG-ML91+-12-F-dG4s is the best model except for human-mtDNA and nDNA. The best model is JTT-ML91+-12-F-dG4s for human-mtDNA and WAG-ML91+-5-F-dG4r for nDNA. The models with the variation of mutation rate (dG4r) and with a uniform substitution rate over sites are shown by cross and plus marks, respectively. The model with the variation of selection constraint (dG4s) is shown by the middle dotted line. The dotted lines in each figure are ones connecting the origin and the respective estimates for the longest branch on the abscissa. In the models specified with the suffix “-5-” for human-mtDNA and nDNA, five parameters were optimized with 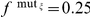 and 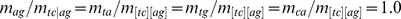.

In Fig. 4, branch lengths estimated by the models with a uniform substitution rate, with the variation of selective constraint, and with the variation of mutation rate over sites are plotted against those estimated by the model with the variation of selective constraint over sites. The dotted lines in these figures are ones connecting the origin and the point of the longest branch on the abscissa. Assuming the variation of mutation rate or selective constraint leads to longer estimates of branch lengths than the uniform substitution rate over sites. However, the estimates of branch lengths are significantly different between the schemes of variable mutation rates and of variable selective constraints over sites, and assuming the variation of mutation rate estimates branch lengths much longer for all datasets than the variation of selective constraint.

Branch lengths estimated by the models with the variation of mutation rate (dG4r) and with a uniform substitution rate are both roughly proportional to those shown on the abscissa, i.e., those estimated by the model with the variation of selective constraint (dG4s). However, as pointed out by Yang et al. [33], a systematic bias in the estimation of branch length is shown; the ratio of the branch length estimate of a worse model to that of the best model tends to be smaller for longer branches irrespective of overestimation or underestimation. For cpDNA-9, cpDNA-55 and mammalian-mtDNA, for which the dG4s is the best model, plus marks for a uniform substitution rate and cross marks for the dG4r are plotted in a concave pattern, although the concave pattern for dG4r is not clear in cpDNA-55. For nDNA, which the dG4r fits better than the dG4s, cross marks for the dG4r are plotted in a slightly convex pattern. This systematic bias indicates that the worse models tend to underestimate the frequencies of multiple substitutions in long branches in comparison with short branches.

**Figure 5:**
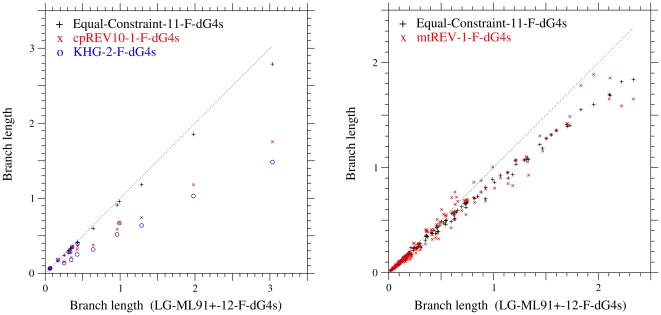
The estimates of branch lengths for the phylogenetic tree of each dataset under the different types of models. Branch lengths estimated for cpDNA-9 (A) and mammalian-mtDNA (B) are compared among models. The abscissa shows the branch lengths estimated by the best model with the variation of selective constraint, LG-ML91+-12-F-dG4s. The dotted line in each figure shows branch lengths estimated by the best model shown on the abscissa.

When the different types of models are compared with each other, the correlation of branch lengths between the models is not always good. In Fig. 5, the estimates of branch lengths for cpDNA-9 and mammalian-mtDNA in the Equal-constraint model and in the amino acid substitution model converted into the codon models are plotted against those in the best model. These estimates for cpDNA-9 are roughly proportional to those in the best model, although there is a systematic bias. However, the correlation of branch lengths between the mtREV-1-F-dG4s and the best model for mammalian-mtDNA is not as good as those between the models for cpDNA-9.

In the result, except for the datasets consisting of highly-homologous sequences, the variation of selective constraint is a better model than the variation of mutation rate, and assuming the variation of mutation rate leads to the overestimation of branch length. Even for highly-homologous sequence families, the model with the variation of selective constraint may not be too bad, because the differences of AIC and BIC between the models with variable mutation rates and with variable selective constraints are not significantly large, and the branch lengths estimated by those models are almost proportional to each other.

### Multiple nucleotide changes in infinitesimal time

Codon substitutions requiring multiple nucleotide changes can be caused by either multiple steps of single nucleotide changes or single steps of multiple nucleotide changes. In the present mechanistic codon substitution model, codon mutations by multiple nucleotide changes in infinitesimal time are taken into account. The mechanistic codon substitution models with the various selective constraint matrices all indicate 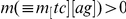 for the datasets cpDNA-9, cpDNA-55, and mammalian-mtDNA, which include long branches. The 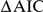 and 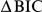 values consistently indicate that the model LG-ML91+-12-F-dG4s, in which multiple nucleotide changes are allowed, fits these datasets better than the model LG-ML91+-11s-F-dG4s, in which multiple nucleotide changes are disallowed. Also, the LRTs for LG-ML91+-11s-F-dG4s nested by LG-ML91+-12-F-dG4s reject the assumption of single nucleotide changes with 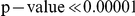 for these datasets; see Tables 2, 3, 4. This result is consistent with a report [19] that the mechanistic codon model could not well fit observed substitution frequency data unless multiple nucleotide changes in infinitesimal time are allowed.

On the other hand, the parameter for multiple nucleotide changes is not significant for the datasets human-mtDNA and nDNA that consist of closely-related or highly-conserved sequences, and whose phylogenetic trees consist of short branches only. This fact indicates that multiple nucleotide changes rarely occur in short evolutionary periods, and multiple nucleotide changes detected in relatively long branches of cpDNA-9, cpDNA-55, and mammalian-mtDNA may result from compensatory substitutions that shortly succeed single nucleotide substitutions, or other mechanisms. A possibility of successive single compensatory substitutions for multiple nucleotide changes was pointed out by Bazykin et al. [26]. Whatever results in multiple nucleotide changes in long evolutionary periods, the present method, in which multiple nucleotide changes in infinitesimal time are allowed, for codon substitutions is effective to improve the likelihood of a phylogenetic tree with long branches.

### Variation of mutation rate over time

The site-specific variation of amino acid substitution rate over time was first discussed as a covarion model by Fitch and Markowitz [35], and recently its significance have been indicated again for rRNA [36] and cytochrome *b* [37]. Although amino acid substitutions may occur in a concerted manner with other interacting sites, causing the variation of selective constraint over time, here we has examined the variation of mutation rate over time at each site.

The present model includes a parameter 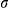 for the variation of mutation rate over time. The scale factor 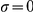 for a 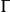 distribution means no variation of mutation rate over time. Because the present simple approximation works by replacing the substitution matrix 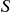 by its expected value 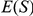 under rate variations, the parameter 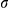 will not only reflect the variation of mutation rate over time but also be affected by the variations of selective constraints over time and of substitution rate over sites, especially if both the variations of mutation rate and of selective constraint over sites are not taken into account; it tends to take larger values in models assuming a uniform rate over sites than variable mutation rates or selective constraints. Also, if only single nucleotide changes in infinitesimal time are assumed, i.e., 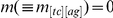, this parameter (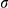) will be estimated to be larger to increase the probability of multiple steps of substitutions. The reverse is also true.

The mechanistic codon substitution models specified with a suffix dG4s, in which selective constraints are variable across sites, all indicate 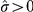 for the datasets cpDNA-9, cpDNA-55, and mammalian-mtDNA, which include long branches. The 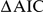 and 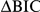 values indicate that the model LG-ML91+-12-F-dG4s including 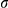 as a parameter fits these datasets better than the model LG-ML91+-11-F-dG4s assuming 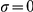. Also, the LRTs for LG-ML91+-11-F-dG4s nested by LG-ML91+-12-F-dG4s reject a constant mutation rate over time with 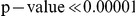 for all cpDNA-9, cpDNA-55, and mammalian-mtDNA; see Tables 2, 3, 4. Therefore, rate variation over time should not be ignored for highly-diverged sequences. The ML estimate of 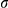 for mammalian-mtDNA is larger than 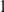, while it is less than 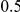 for the other two datasets. The variation of mutation rate among lineages in primate mtDNAs has been indicated [6], [55].

As shown in Tables 2, 3, 4, 5, 6, when mutation rates are assumed to be variable across sites, i.e., in the mechanistic codon substitution models specified with a suffix dG4r, the parameter 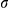 has been estimated to be almost equal to zero for all the datasets, even for the datasets cpDNA-9, cpDNA-55, and mammalian-mtDNA, for which the models assuming variable selective constraints indicate 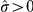. Variable mutation rates across sites are taken into account in such a way that each site has multiple mutation rates with certain probabilities given by a discrete gamma distribution. Thus, in the present approximation it would be hard to distinguish the variation of mutation rate over time at each site from that over sites in these models.

### Transition/transversion bias

One of the advantages in mechanistic codon substitution models over amino acid substitution models is that mutational tendencies at the nucleotide level can be estimated. The estimation of mutational tendencies by mechanistic codon substitution models must be more precise than by nucleotide substitution models applied to all codon positions, because selection at the amino acid level is taken into account.

Transitional substitutions have been noted to occur more frequently than transversions [56], [57], and transition/transversion rate bias is more pronounced in animal mitochondrial DNAs than in nuclear or chloroplast DNAs [58]. Different measures have been used for transition to transversion bias [55], [58], [59]. One is the ratio of transitional differences to transversional differences between two sequences. Another is the ratio of the total transitional to the total transversional rate. Also, the ratio of transitional to transversional substitution exchangeability has been used. Here, the ratio of the mean transitional to the mean transversional exchangeability is used, because each type of transitional and transversional mutations occurs with a different exchangeability. The ratio (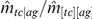) of the mean transitional to the mean transversional exchangeability is listed in Tables 2, 3, 4, 5, 6 for all datasets. The values of 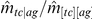 in the mechanistic codon substitution models with the various estimates of selective constraints fall into a narrow range for each dataset. They range from 3.7 to 7.2 for mammal-mtDNA, and from 30.5 to 39.0 for human-mtDNA. On the other hand, they fall into the range of much smaller values from 1.7 to 2.4 for cpDNA-9, from 2.4 to 2.7 for cpDNA-55, and from 2.3 to 2.8 for nDNA. The ratio of the mean transitional to the mean transversional exchangeability is estimated to be almost 10–20 times larger for human mitochondrial DNA but only 2–3 times larger for mammalian mitochondrial DNA than for nuclear and chloroplast DNAs. Adachi and Hasegawa [6] reported that the transitional mutation rate and the ratio of transitional to transversional mutation rate at four-fold degenerate sites of mtDNA were higher by about two times in humans than in apes. On the other hand, Yang and Yoder [55] showed that the maximum likelihood estimate of the ratio of transitional to transversional substitution rate changes with the species included in the analysis, and was always larger at low than at high sequence divergence. It was suggested [55] that the variable rates of transitional and transversional mutations among evolutionary lineages might cause such a sample dependence.

### Conclusions

In the present mechanistic codon substitution model, single nucleotide mutations are modeled by the GTR model and multiple nucleotide mutations in infinitesimal time are assumed to occur independently at each position of codon, and selective constraints on amino acids are approximated by a linear function of the empirical selective constraints. It has been shown that even the Equal-Constraint model performs far better for a wide range of sequences from highly-homologous to highly-diverged sequences than both the No-Constraint model and the amino acid substitution models converted into the codon substitution models. The No-Constraint model is a nucleotide substitution model extended to allow multiple nucleotide changes in infinitesimal time. On the other hand, the codon substitution model converted from the amino acid substitution model is extended here in such a way that the special case of 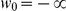 is exactly equivalent to the amino acid substitution model [17]. Thus, the performance of the Equal-Constraint model indicates that codon substitution models are superior to nucleotide and amino acid substitution models.

The present analyses have also shown that the mechanistic models with the amino acid dependent selective constraints do not only perform far better especially for phylogenetic trees consisting of relatively long branches than the Equal-Constraint model, but better even for phylogenetic trees consisting of short branches. This result indicates the superiority of the selective constraint matrices 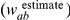 estimated by maximizing the respective likelihoods of the observed substitution frequency matrices of 1-PAM [19]. In long branches, nonsynonymous substitutions increase, and therefore the proper evaluation of selective constraints on amino acids becomes critical. On the other hand, in short branches in which nonsynonymous substitutions are insignificant, the proper evaluation of mutational tendencies at the nucleotide level becomes important. The former is the situation in which amino acid substitution models perform better than nucleotide substitution models. Inversely, the latter is the situation in which nucleotide substitution models perform better, although they are not superior for slow-evolving proteins, because there is a possibility that synonymous substitutions are saturated even in short branches; the dataset nDNA is an example of such a case. However, mutational tendencies at the nucleotide level and the strength of selective constraints cannot be tailored to each gene in the amino acid substitution models, and selection on amino acid replacements cannot be taken into account in the nucleotide substitution models. Thus, mechanistic codon models that can tailor both mutational tendencies and the strength of selective constraints are superior to both nucleotide and amino acid substitution models.

It was pointed out [18] that codon substitution models require intensive computation to recalculate eigenvalues and eigenvectors of a 64-dimensional matrix. Simultaneous optimizations of a tree topology and model parameters may be hard. However, model parameters may be fixed at the values estimated for one of the reasonable trees, because the optimum values of model parameters do not severely depend on a tree topology, unless tree topologies are unrealistic. On the other hand, the mechanistic codon substitution model can provide much information on mutational tendencies and the strength of selective constraints. In addition, the present model enables us to distinguish the variations of mutation rate and of selective constraint over sites. The variation of mutation rate over time can also be discussed.

The present analyses show that multiple nucleotide changes in infinitesimal time are statistically significant in long branches as well as the variation of mutation rate over time. It has been also shown that the variation of amino acid substitution rate over sites results from variable selective constraints rather than variable mutation rates at least in the phylogenetic trees of cpDNA-9, cpDNA-55, and mammalian-mtDNA including long branches. Branch lengths will be overestimated for these datasets if the variation of mutation rate over sites is assumed instead of the variation of selective constraint. The capability of the mechanistic codon substitution models to extract biological knowledge from protein-coding sequences makes them superior to both nucleotide and amino acid substitution models.
